# Mortality and morbidity in community-acquired sepsis in European pediatric intensive care units: a prospective cohort study from the European Childhood Life-threatening Infectious Disease Study (EUCLIDS)

**DOI:** 10.1186/s13054-018-2052-7

**Published:** 2018-05-31

**Authors:** Navin P. Boeddha, Luregn J. Schlapbach, Gertjan J. Driessen, Jethro A. Herberg, Irene Rivero-Calle, Miriam Cebey-López, Daniela S. Klobassa, Ria Philipsen, Ronald de Groot, David P. Inwald, Simon Nadel, Stéphane Paulus, Eleanor Pinnock, Fatou Secka, Suzanne T. Anderson, Rachel S. Agbeko, Christoph Berger, Colin G. Fink, Enitan D. Carrol, Werner Zenz, Michael Levin, Michiel van der Flier, Federico Martinón-Torres, Jan A. Hazelzet, Marieke Emonts, on behalf of the EUCLIDS consortium

**Affiliations:** 1 Intensive Care and Department of Pediatric Surgery, Erasmus MC-Sophia Children’s Hospital, University Medical Center Rotterdam, Wytemaweg 80, 3015 CN Rotterdam, The Netherlands; 2 Department of Pediatrics, Division of Pediatric Infectious Diseases & Immunology, Erasmus MC-Sophia Children’s Hospital, University Medical Center Rotterdam, Wytemaweg 80, 3015 CN Rotterdam, The Netherlands; 3 Faculty of Medicine, The University of Queensland, St Lucia Queensland, Brisbane, 4072 Australia; 4 Paediatric Critical Care Research Group, Mater Research Institute, The University of Queensland, Aubigny Place, Raymond Terrace, Brisbane, Australia; 5 Paediatric Intensive Care Unit, Lady Cilento Children’s Hospital, Children’s Health Queensland, 501 Stanley St, Brisbane, Australia; 6 Department of Pediatrics, Bern University Hospital, Inselspital, University of Bern, Freiburgstrasse 8, 3010 Bern, Switzerland; 7 Department of Paediatrics, Juliana Children’s Hospital/Haga Teaching Hospital, Els Borst-Eilersplein 275, 2545 AA The Hague, The Netherlands; 8 Section of Pediatrics, Imperial College London, Level 2, Faculty Building South Kensington Campus, London, SW7 2AZ UK; 9 Translational Pediatrics and Infectious Diseases Section- Pediatrics Department, Hospital Clínico Universitario de Santiago de Compostela, Travesía da Choupana, 15706 Santiago de Compostela, Spain; 10 Genetics- Vaccines- Infectious Diseases and Pediatrics research group GENVIP, Health Research Institute of Santiago IDIS/SERGAS, Travesía da Choupana, 15706 Santiago de Compostela, Spain; 11 Department of General Paediatrics, Medical University of Graz, Auenbruggerplatz 34/2, A-8036 Graz, Austria; 12 Radboudumc Technology Center Clinical Studies, Radboudumc, Geert Grooteplein Zuid 10, 6525 GA Nijmegen, The Netherlands; 13 Section of Pediatric Infectious Diseases, Laboratory of Medical Immunology, Radboud Institute for Molecular Life Sciences, Radboudumc, Geert Grooteplein Zuid 10, 6525 GA Nijmegen, The Netherlands; 14 Radboud Center for Infectious Diseases, Radboudumc, Geert Grooteplein Zuid 10, 6525 GA Nijmegen, The Netherlands; 15 Department of Paediatrics, Faculty of Medicine, Imperial College London, South Kensington Campus, London, SW7 2AZ UK; 16 St Mary’s Hospital, Imperial College Healthcare NHS Trust, Praed Street, London, W2 1NY UK; 17 Division of Paediatric Infectious Diseases, Alder Hey Children’s NHS Foundation Trust, Eaton Rd, Liverpool, L12 2AP UK; 18 Institute of Infection & Global Health, University of Liverpool, 8 West Derby St, Liverpool, L7 3EA UK; 19 Micropathology Ltd, University of Warwick Science Park, Venture Centre, Sir William Lyons Road, Coventry, CV4 7EZ UK; 20 Medical research Council Unit, Atlantic Boulevard, Fajara, P. O. Box 273, Banjul, The Gambia; 21 Department of Paediatric Intensive Care, Great North Children’s Hospital, Newcastle upon Tyne Hospitals NHS Foundation Trust, Victoria Wing, Royal Victoria Infirmary, Newcastle upon Tyne, NE1 4LP UK; 22 Institute of Cellular Medicine, Newcastle University, 4th Floor, William Leech Building, Framlington Place, Newcastle upon Tyne, NE2 4HH UK; 23 Division of Infectious Diseases and Hospital Epidemiology, and Children’s Research Center, University Children’s Hospital Zurich, Steinwiesenstrasse 75, 8032 Zurich, Switzerland; 24 Pediatric Infectious Diseases and Immunology Amalia Children’s Hospital, and Radboudumc Expertise Center for Immunodeficiency and Autoinflammation (REIA), Radboudumc, Geert Grooteplein Zuid 10, 6525 GA Nijmegen, The Netherlands; 25 Department of Public Health, Erasmus MC, University Medical Center Rotterdam, Wytemaweg 80, 3015 CN Rotterdam, The Netherlands; 26 Paediatric Infectious Diseases and Immunology Department, Great North Children’s Hospital, Newcastle upon Tyne Hospitals NHS Foundation Trust, Victoria Wing, Royal Victoria Infirmary, Newcastle upon Tyne, NE1 4LP UK; 27 NIHR Newcastle Biomedical Research Centre based at Newcastle upon Tyne Hospitals NHS Trust and Newcastle University, Westgate Rd, Newcastle upon Tyne, NE4 5PL UK

**Keywords:** Bacteremia, Meningococcal infections, Pneumococcal infections, Mortality, Morbidity

## Abstract

**Background:**

Sepsis is one of the main reasons for non-elective admission to pediatric intensive care units (PICUs), but little is known about determinants influencing outcome. We characterized children admitted with community-acquired sepsis to European PICUs and studied risk factors for mortality and disability.

**Methods:**

Data were collected within the collaborative Seventh Framework Programme (FP7)-funded EUCLIDS study, which is a prospective multicenter cohort study aiming to evaluate genetic determinants of susceptibility and/or severity in sepsis. This report includes 795 children admitted with community-acquired sepsis to 52 PICUs from seven European countries between July 2012 and January 2016. The primary outcome measure was in-hospital death. Secondary outcome measures were PICU-free days censured at day 28, hospital length of stay, and disability. Independent predictors were identified by multivariate regression analysis.

**Results:**

Patients most commonly presented clinically with sepsis without a source (*n* = 278, 35%), meningitis/encephalitis (*n* = 182, 23%), or pneumonia (*n* = 149, 19%). Of 428 (54%) patients with confirmed bacterial infection, *Neisseria meningitidis* (*n* = 131, 31%) and *Streptococcus pneumoniae* (*n* = 78, 18%) were the main pathogens. Mortality was 6% (51/795), increasing to 10% in the presence of septic shock (45/466). Of the survivors, 31% were discharged with disability, including 24% of previously healthy children who survived with disability. Mortality and disability were independently associated with *S. pneumoniae* infections (mortality OR 4.1, 95% CI 1.1–16.0, *P* = 0.04; disability OR 5.4, 95% CI 1.8–15.8, *P* < 0.01) and illness severity as measured by Pediatric Index of Mortality (PIM2) score (mortality OR 2.8, 95% CI 1.3–6.1, *P* < 0.01; disability OR 3.4, 95% CI 1.8–6.4, *P* < 0.001).

**Conclusions:**

Despite widespread immunization campaigns, invasive bacterial disease remains responsible for substantial morbidity and mortality in critically ill children in high-income countries. Almost one third of sepsis survivors admitted to the PICU were discharged with some disability. More research is required to delineate the long-term outcome of pediatric sepsis and to identify interventional targets. Our findings emphasize the importance of improved early sepsis-recognition programs to address the high burden of disease.

**Electronic supplementary material:**

The online version of this article (10.1186/s13054-018-2052-7) contains supplementary material, which is available to authorized users.

## Background

Pediatric sepsis represents one of the most common reasons for pediatric intensive care unit (PICU) admission, and the prevalence and mortality in high-income countries has become comparable to that in adults [1–5]. In 2013, 10% of childhood deaths under the age of 5 years in high-income countries were attributable to infections, with the majority of acute infection-related deaths occurring in PICUs [6]. Recent reports have demonstrated the major impact of comorbidities with increasing rates of healthcare-associated infections [1, 2, 4, 5, 7–9].

In contrast, recent data on community-acquired sepsis are limited. Community-acquired sepsis represents specific patterns, affecting different hosts, and involving different pathogens, which may translate into different outcomes compared to healthcare-associated infections [10, 11]. In view of the need to develop improved strategies for early recognition and treatment of sepsis, as demanded by the recent resolution of the World Health Organization [12], it is imperative to assess contemporary characteristics of epidemiology and severity predictors for community-acquired sepsis [13]. Previous larger epidemiological sepsis studies have been predominantly based on hospital coding or PICU databases with mortality as the main outcome [1, 5]. A recent roadmap for future sepsis research highlighted the inherent limitations of such approaches, identifying the need to define the longer-term impact on survivors [14]. While increasing evidence in neonatal and adult patients demonstrates that new cognitive impairment, functional disability, and impaired quality of life are common amongst sepsis survivors [15–18], little is known about disability in pediatric sepsis survivors [19, 20].

The aim of this study was to characterize the clinical presentation, pathogens, mortality, and disability in children admitted to European PICUs with community-acquired sepsis, based on patients recruited through the multinational prospective European Childhood Life-threatening Infectious Disease Study (EUCLIDS).

## Methods

### Consortium and study sites

The EUCLIDS is a Seventh Framework Programme (FP7) project in the context of the European Union’s Research and Innovation funding program for 2007–2013. This large-scale prospective, multicenter, cohort study aimed to identify genes, and biological pathways that determine susceptibility and severity in life-threatening bacterial infections of childhood. The EUCLIDS clinical network includes predominantly academic pediatric hospitals that host a total of 52 PICUs from 7 European countries; Austria (9), Germany (7), Lithuania (1), The Netherlands (5), Spain (9), Switzerland (8), and the UK (13).

### Study patients

From July 2012 to January 2016, patients aged 29 days to 18 years admitted with community-acquired sepsis to PICUs in participating centers were prospectively enrolled in the study. The 2005 pediatric consensus criteria for sepsis were used, dividing patients into those with sepsis, severe sepsis, or septic shock [21]. Healthcare-associated infections [22], patients undergoing bone marrow transplant, and patients already recruited who were readmitted within the same illness episode were excluded. Children with a central venous catheter at admission were not excluded. Although the consortium was specifically interested in patients with invasive meningococcal, pneumococcal, staphylococcal, salmonella, and group A streptococcal infections, representing the most common causes of community-acquired sepsis in children, patients with illness due to other organisms were included as well. Patients were recruited as early as possible in the illness within a time window from presentation to the time when culture results became available.

### Ethical aspects

This study was conducted in accordance with the Declaration of Helsinki and Good Clinical Practice guidelines. The study protocol was approved by at least one ethical review board in every country (Coordinating Center Research Ethics Committee reference: 11/LO/1982) [23]. Written informed consent was obtained from parents or legal guardians. In the Swiss study [24, 25], consent was obtained for collection of blood for research, but waiver of consent for collection of anonymized epidemiological data was approved.

### Clinical data collection

Data on clinical presentation, underlying disease, illness severity, management, microbiological results, and outcome were collected prospectively. Children were split into four age categories; infants (29 days to < 1 year), toddlers (≥ 1 year to < 5 years), school-aged children (≥ 5 years to < 12 years), and adolescents (≥ 12 years to < 18 years). Underlying conditions at admission to the PICU were classified following the pediatric complex chronic conditions classification system [26]. Illness severity was measured by the Pediatric risk of mortality score (PRISM) [27] and Pediatric Index of Mortality (PIM2) [28]. We studied lactate values obtained on admission, concomitant with PIM2 data collection. Invasive bacterial infections were defined as isolation by culture or PCR of a bacterial organism from a normally sterile site. We considered blood, cerebrospinal fluid, urine, bronchoalveolar lavage, joint aspirate, abscess aspirate, intraoperative swabs, and pleural aspirate as sterile sites. Urine positive for pneumococcal antigen was also considered as an invasive bacterial infection if patients met sepsis criteria. Positive cultures from sites such as endotracheal tube aspirate, nasopharyngeal aspirate, throat/nasal swabs, and wounds were not considered as sterile sites. We defined potentially vaccine-preventable infections as infections caused by pathogens that are included in currently available national immunization programs, with a focus on *Haemophilus influenzae* type B (HiB), meningococcus serogroups ACWY (MenACWY), meningococcus serogroup B (MenB), meningococcus serogroup C (MenC), pneumococcal conjugate vaccine 7 (PCV7, Prevnar, serotypes 4, 6B, 9V, 14, 18C, 19F and 23F), pneumococcal conjugate vaccine 10 (PCV10, Synflorix, additional serotypes 1, 5, 7F), pneumococcal conjugate vaccine 13 (PCV13, Prevnar 13, additional serotypes 3, 6A, 19A) and pneumococcal polysaccharide vaccine 23 (PPSV23, additional serotypes 2, 8, 9N, 10A, 11A, 12F, 15B, 17F, 20, 22F, 33F). Data on routine immunization schedules and uptake in the countries involved are presented in Additional file 1: Table S1. We classified patients as primary bloodstream infection and sepsis without a known source (grouped as *no focus*) versus patients with a clinical focus of infection. Patients admitted with systemic inflammatory response syndrome (SIRS) in the presence of suspected infection (i.e. sepsis), in whom a bacterial, viral, or fungal infection eventually could not be confirmed, were categorized as clinical presentation *other*.

### Outcomes

The primary outcome measure was death in hospital, recorded as alive or death status at the time of hospital discharge. Secondary outcomes were assessed at time of hospital discharge and included disability, PICU-free days censored at day 28 (days alive and free from the need for intensive care), and hospital length of stay. Disability was defined as a Pediatric Overall Performance Category (POPC) scale > 1 [29], need for skin graft, amputation, or hearing loss. The POPC scale was determined either by direct observation or by chart review and ranges from 1 to 6: (1) good overall performance, (2) mild overall disability, (3) moderate overall disability, (4) severe overall disability, (5) coma or vegetative state, and (6) brain death. A description of these categories is presented in Additional file 1: Table S2 [29]. PICU-free days in patients who died were considered zero. All data were collected in web-based case report forms. Monthly telephone conferences, biannual meetings, clinical protocols including case definitions, data audits, and monitoring, ensured uniform procedures among study sites.

### Statistical analysis

Categorical variables are presented as counts (percentages). We used the chi-Square test or Fisher’s exact test to compare frequency distributions between two categorical variables. Post-hoc Bonferroni correction for multiple testing was applied when we compared age groups with features on clinical presentation or pathogens. Continuous variables are presented either as mean (± standard deviation (SD)) for data with a parametric distribution or as median (interquartile range (IQR)) for non-parametric data. We tested differences between groups with analysis of variance (ANOVA) or Kruskal-Wallis and Student’s *t* test, or Mann-Whitney U test, as appropriate. Logistic regression (of binary outcome measures) and linear regression (of continuous outcome measures) were used to identify independent predictors. Variables with a *P* value <0.20 in the univariable analysis were included in the multivariable analysis. In the multivariable analysis, we only included one parameter of illness severity (PIM2), because of multicollinearity of the illness severity parameters. Area under the receiver operating characteristic (AUROC) curve analysis was applied to determine the Youden index. Sensitivity, specificity, positive predictive value (PPV), negative predictive value (NPV), positive likelihood ratio (PLR), and negative likelihood ratio (NLR) were calculated for the optimal cutoff value of lactate. Statistical analyses were performed with SPSS version 21 (Armonk, USA). Graphs were created in GraphPad Prism 5.00. A *P* value <0.05 was considered statistically significant.

## Results

**Fig. 1:**
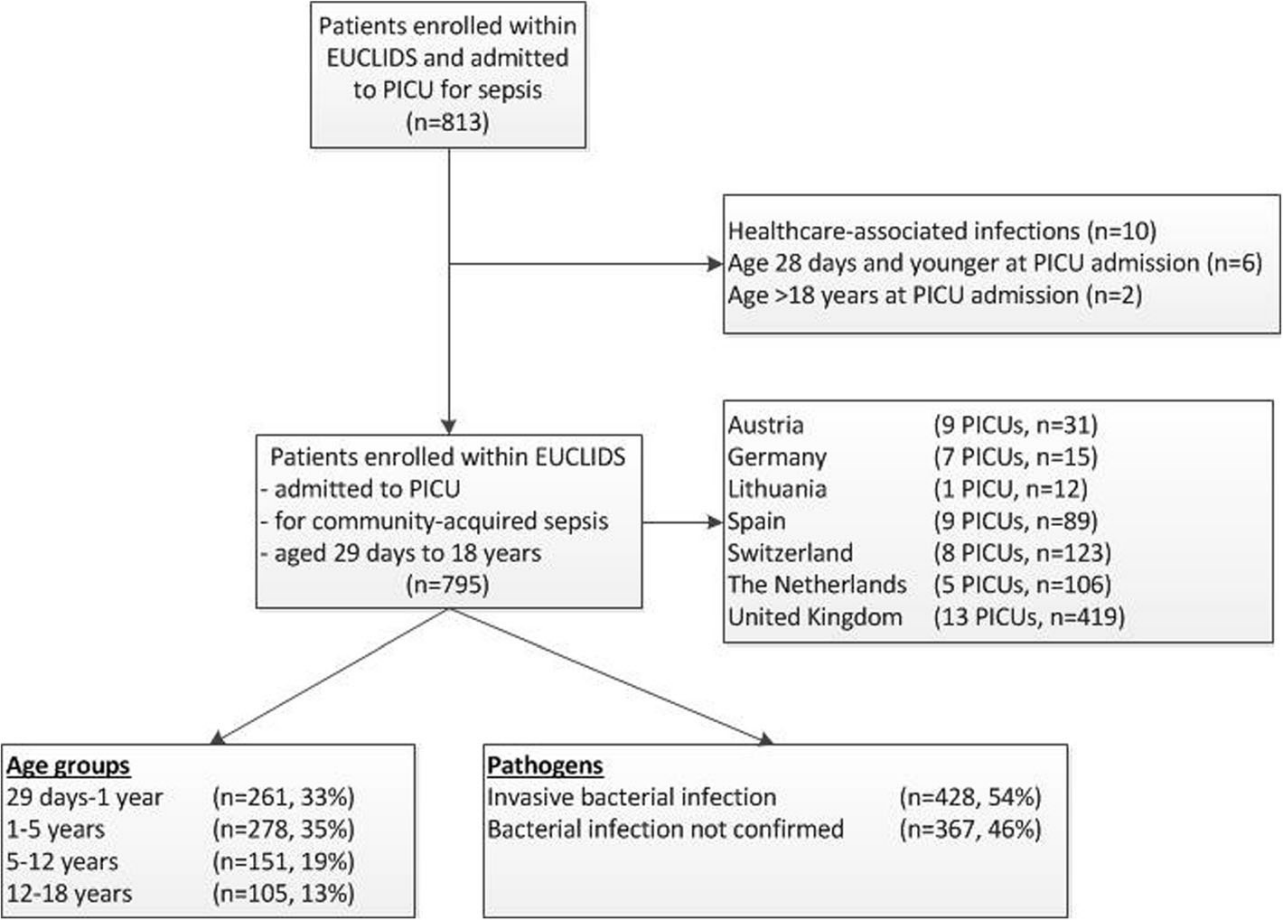
Patients admitted with community-acquired sepsis to European pediatric intensive care units (PICUs). EUCLIDS, European Childhood Life-threatening Infectious Disease Study

From July 2012 to January 2016, 795 children (54% male, median age 2.2 years (IQR 8 months to 6 years)) admitted with community-acquired sepsis to 52 PICUs in 7 European countries were enrolled (Fig. 1). Baseline characteristics by age category are presented in Table 1. An underlying condition was present in 288 patients (36%), of which prematurity and neonatal conditions (*n* = 87, 11%) and neurologic and neuromuscular conditions were most common (*n* = 70, 9%). A total of 466 patients (59%) presented with septic shock.

**Table 1:** Baseline characteristics of children admitted with community-acquired sepsis to PICU

	All patients (*n* = 795)	29 days–12 months (*n* = 261)	1–5 years (*n* = 278)	5–12 years (*n* = 151)	12–18 years (*n* = 105)	*P*
Sex (male *n*, %)	428 (54%)	151 (58%)	148 (53%)	76 (50%)	53 (51%)	ns
Age	2 years (8 months–6 years)	5 months (2–8 months)	2 years (18 months–3 years)	8 years (6–10 years)	15 years (14–16 years)	–
Ethnicity^a^						ns
African/North African	46 (6%)	15 (6%)	12 (4%)	14 (10%)	5 (5%)	
Asian	57 (7%)	22 (9%)	18 (7%)	10 (7%)	7 (7%)	
European	607 (79%)	195 (76%)	222 (82%)	106 (75%)	84 (82%)	
Meso/South American	7 (1%)	1 (0%)	3 (1%)	1 (1%)	2 (2%)	
Middle Eastern	10 (1%)	5 (2%)	1 (0%)	2 (1%)	2 (2%)	
Other/mixed	45 (6%)	18 (7%)	15 (6%)	9 (6%)	3 (3%)	
Time interval onset symptoms to hospital admission^b^ (days)	1 (1–3)	1 (0–3)	2 (1–)	2 (0–4)	1 (1–3)	ns
Immunizations up to date^c^	585 (89%)	177 (82%)	219 (89%)	114 (95%)	75 (97%)	< 0.001
Number of underlying conditions						< 0.01
None	507 (64%)	176 (67%)	192 (69%)	85 (56%)	54 (51%)	
1	175 (22%)	55 (21%)	53 (19%)	36 (24%)	31 (30%)	
≥ 2	113 (14%)	30 (12%)	33 (12%)	30 (20%)	20 (19%)	
Underlying conditions						< 0.01
Neurologic and neuromuscular	70 (9%)	6 (2%)	17 (6%)	27 (18%)	20 (19%)	
Cardiovascular	52 (7%)	14 (5%)	27 (10%)	8 (5%)	3 (3%)	
Respiratory	40 (5%)	14 (5%)	12 (4%)	9 (6%)	5 (5%)	
Renal and urologic	18 (2%)	5 (2%)	8 (3%)	2 (1%)	3 (3%)	
Gastrointestinal	35 (4%)	9 (3%)	11 (4%)	8 (5%)	7 (7%)	
Hematologic or immunologic	11 (1%)	2 (0%)	4 (1%)	4 (3%)	1 (1%)	
Metabolic	20 (3%)	5 (2%)	6 (2%)	5 (3%)	4 (4%)	
Other congenital or genetic defect	56 (7%)	9 (3%)	18 (6%)	17 (11%)	12 (11%)	
Malignancy	9 (1%)	1 (0%)	1 (0%)	2 (1%)	5 (5%)	
Premature and neonatal	87 (11%)	56 (21%)	19 (7%)	9 (6%)	3 (3%)	
Other	52 (7%)	10 (4%)	16 (6%)	14 (9%)	12 (11%)	
Illness severity						
PRISM score^d^	14 (7–21)	14 (7–22)	15 (8–22)	14 (8–21)	11 (4–16)	< 0.01
PIM2 score^e^ (predicted death, %)	4.0 (1.1–9.5)	4.0 (1.0–8.2)	4.8 (1.1–10.3)	4.2 (1.1–17.1)	3.3 (0.9–9.0)	ns
Lactate at PICU admission^f^ (mmol/L)	1.8 (1.1–3.4)	1.6 (1.0–3.2)	1.6 (1.0–3.2)	2.2 (1.1–3.7)	2.3 (1.2–4.7)	ns
Septic shock (*n*, %)	466 (59%)	133 (51%)	168 (60%)	94 (62%)	71 (68%)	< 0.05

Values are reported as counts (percentages) or medians (interquartile ranges), unless stated otherwise

*Abbreviations: PICU* pediatric ICU, *PRISM* Pediatric Risk of Mortality [27], *PIM2* Pediatric Index of Mortality 2 [28], *ns* not significant

^a^Ethnicity data were available for 772/795 patients; 256/261 infants, 271/278 toddlers, 142/151 school-aged children, and 103/105 adolescents

^b^Time interval from onset of symptoms to hospital admission was available for 642/795 patients; 212/261 infants, 229/278 toddlers, 111/151 school-aged children, and 90/105 adolescents

^c^Immunization data were available for 657/795 patients; 215/261 infants, 245/278 toddlers, 120/151 school-aged children, and 77/105 adolescents

^d^PRISM score was available for 672/795 patients; 223/261 infants, 240/278 toddlers, 118/151 school-aged children, and 91/105 adolescents

^e^PIM2 score was available for 681/795 patients; 224/261 infants, 243/278 toddlers, 123/151 school-aged children, and 91/105 adolescents

^f^Lactate at PICU admission was available for 444/795 patients; 146/261 infants, 167/278 toddlers, 76/151 school-aged children, and 55/105 adolescents

### Clinical presentations and pathogens

Primary bloodstream infection and sepsis without a known source among patients with community-acquired sepsis accounted for 278 (35%) admissions to the PICU. The other most common clinical illnesses were meningitis/encephalitis (*n* = 182, 23%) and pneumonia (*n* = 149, 19%) (Additional file 1: Figure S1). Clinical presentation were similar across age groups, apart from osteomyelitis/septic arthritis, which was diagnosed more frequently in school-aged children than in infants (7.3% versus 0.4%, *P* value = 0.002).

**Fig. 2:**
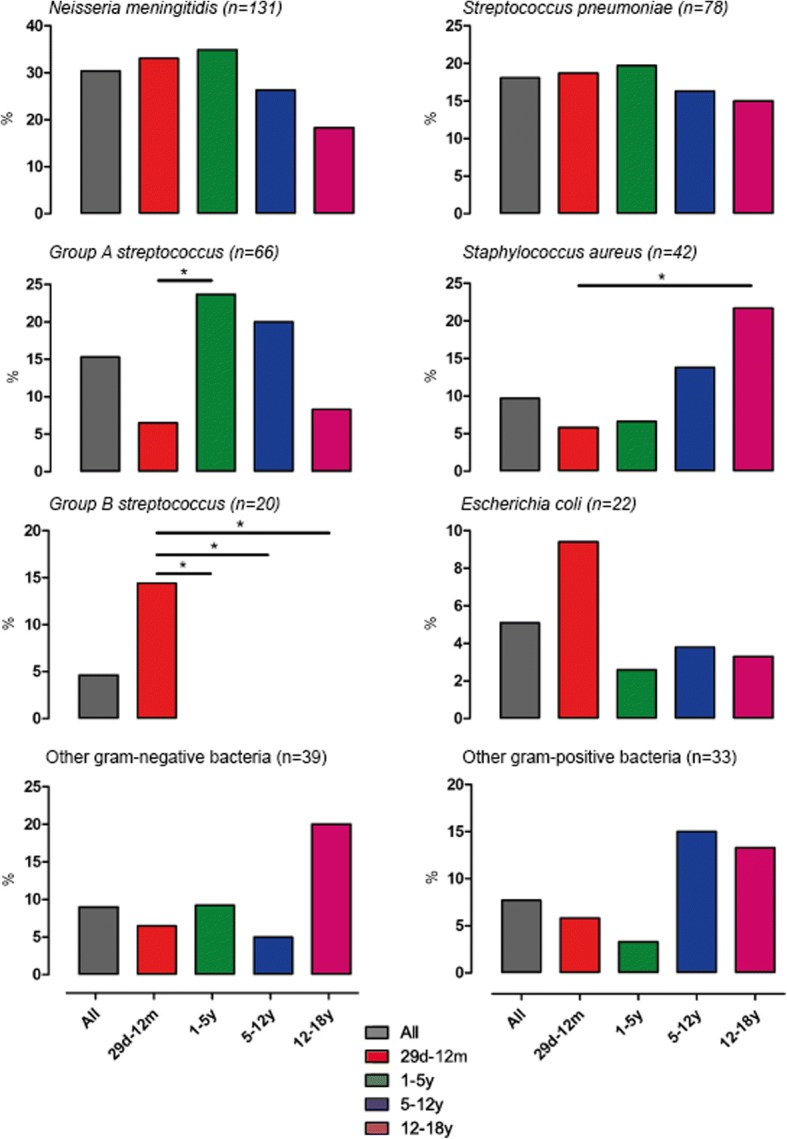
Invasive pathogens in patients with community-acquired sepsis admitted to the pediatric intensive care unit. Invasive pathogens (*n* = 428) by age category. Numbers are higher as three patients had mixed bacterial infections (one patient with *Staphylococcus aureus*/gram-negative bacteria, two patients with gram-negative/gram-positive bacteria). The y-axis represents the percentage of respective pathogen within the age category, i.e. percentages of all pathogens within an age category add up to 100%. d, days; m, months

Bacterial etiology was confirmed in 428 patients (54%), including 334 patients (42%) with a positive blood culture, and pathogen distribution was associated with age (Fig. 2). *Neisseria meningitidis* was the most commonly identified pathogen (*n* = 131, 31%), of which serogroup B was most prevalent (*n* = 89, 68%), followed by *Streptococcus pneumoniae* (*n* = 78, 18%, of which serotypes 3 (n = 7, 14%) and 10A (*n* = 6, 12%) were most commonly identified in those with serotyping information available (*n* = 51)) (Additional file 1: Table S3).

Of the 466 patients with septic shock, an invasive bacterial infection was confirmed in 255 patients (55%). *N. meningitidis* (*n* = 91, 36%) and group A streptococcus (*n* = 49, 19%) were the most commonly identified pathogens, followed by *Streptococcus pneumoniae* (*n* = 33, 13%).

### Therapy

Invasive ventilation was used in 519 patients (69%) (median length of invasive respiratory support 5 days, IQR 3–8, *n* = 43 with missing data) and vasoactive agents for 418 patients (57%) (median 3 days, IQR 2–5 days, *n* = 56 with missing data). Infants needed invasive ventilation more frequently than adolescents (73% versus 58%, *P* value = 0.03).

### Mortality and PICU-free survival

Of the 795 children admitted to PICU with community-acquired sepsis, 51 patients (6%) died. Mortality increased to 10% (*n* = 45) in patients with septic shock. Univariable analysis showed that the presence of bacteremia (odds ratio (OR) 4.4, 95% confidence interval (CI) 2.3–8.4, *P* < 0.001) and infections caused by *S. pneumoniae* (OR 2.5, 95% CI 1.2–5.1, *P* = 0.01) were associated with mortality in patients with sepsis (Table 2). In addition, illness severity, as measured by PRISM score, PIM2 score, invasive ventilation, the need for inotropes, and higher lactate at PICU admission, were also associated with sepsis mortality. The AUROC for lactate as a predictor of mortality was 0.723 (95% CI 0.624–0.822), with an optimal cutoff value of 2.2 mmol/L (sensitivity 0.78, specificity 0.60) (Additional file 1: Figure S2).

**Table 2:** Predictors of death in children with community-acquired sepsis

	Sepsis survivors (*n* = 744)	Deaths (*n* = 51)	Univariable odds ratio for death (95% CI)	*P*	Multivariable odds ratio for death (95% CI)	*P*
Sex						
Male	399/744 (54%)	29/51 (57%)	Reference			
Female	345/744 (46%)	22/51 (43%)	0.9 (0.5–1.6)	0.65	NA	
Age						
29 days–12 months (infants)	248/744 (33%)	13/51 (26%)	Reference			
1–5 years (toddlers)	259/744 (35%)	19/51 (37%)	1.4 (0.7–2.9)	0.37	NA	
5–12 years (school -aged children)	140/744 (19%)	11/51 (22%)	1.5 (0.7–3.4)	0.34	NA	
12–18 years (adolescents)	97/744 (13%)	8/51 (16%)	1.6 (0.6–3.9)	0.33	NA	
Time interval from onset of symptoms to hospital admission^a^ (days)	1 (1–3)	2 (1–4)	1.0 (1.0–1.1)	0.47	NA	
Immunizations up to date						
No	70/621 (11%)	2/36 (6%)	Reference			
Yes	551/621 (89%)	34/36 (94%)	2.2 (0.5–9.2)	0.30	NA	
Underlying condition						
No	479/744 (64%)	28/51 (55%)	Reference			
Yes	265/744 (36%)	23/51 (45%)	1.5 (0.8–2.6)	0.18	0.7 (0.2–2.0)	0.46
Illness severity						
PRISM score^b^	14 (7–20)	22 (15–30)	1.1 (1.0–1.1)	< 0.001	NA	
PIM2 score^c^ (predicted death, %)	3.9 (1.0–9.1)	14.7 (3.8–48.0)	3.9 (2.1–7.2)	< 0.001	2.8 (1.3–6.1)	< 0.01
Lactate at PICU admission^d^ (mmol/L)	1.7 (1.0–3.3)	3.3 (2.3–5.4)	8.9 (2.7–29.1)	< 0.001	NA	
Invasive ventilation	474/705 (67%)	45/47 (96%)	11.0 (2.6–45.6)	0.001	NA	
Inotropes	377/693 (54%)	41/46 (89%)	6.9 (2.7–17.6)	< 0.001	NA	
Bacteremia						
No	445/741 (60%)	13/51 (26%)	Reference			
Yes	296/741 (40%)	38/51 (75%)	4.4 (2.3–8.4)	< 0.001	7.4 (1.0–56.6)	0.06
Clinical syndromes						
No focus	254/744 (34%)	24/51 (47%)	1.7 (1.0–3.0)	0.06	3.0 (0.8–10.9)	0.09
Meningitis/encephalitis	172/744 (23%)	10/51 (20%)	0.8 (0.4–1.6)	0.56	NA	
Pneumonia	138/744 (19%)	11/51 (22%)	1.2 (0.6–2.4)	0.59	NA	
Other focus	180/744 (24%)	6/51 (12%)	0.4 (0.2–1.0)	0.05	1.8 (0.3–11.8)	0.52
Invasive pathogens^e^						
*N*. *meningitidis*	120/386 (31%)	11/39 (28%)	0.8 (0.4–1.8)	0.88	NA	
*S*. *pneumoniae*	65/386 (17%)	13/39 (33%)	2.5 (1.2–5.1)	0.01	4.1 (1.1–16.0)	0.04
Group A streptococcus	60/386 (16%)	6/39 (15%)	1.0 (0.4–2.5)	1.0	NA	
*S. aureus*	37/386 (10%)	4/39 (10%)	1.1 (0.4–3.2)	0.88	NA	
Other invasive pathogen	104/386 (27%)	5/39 (13%)	0.4 (0.2–1.0)	0.06	0.3 (0.0–2.2)	0.21

This study included 795 children admitted with community-acquired sepsis, of whom 51 patients died. Multivariable analysis included variables with a *P* value < 0.20 in univariable analysis. Because parameters of illness severity are strongly correlated, only the Pediatric Index of Mortality 2 (PIM2) [28] score has been included in multivariable analysis. Values are reported as counts (percentages) or medians (interquartile ranges), unless stated otherwise

*PRISM* Pediatric Risk of Mortality [27], *NA* not applicable

^a^Time interval from onset of symptoms to hospital admission was available for 609/744 sepsis survivors and 33/51 non-survivors

^b^PRISM score was available for 636/744 sepsis survivors and 36/51 non-survivors

^c^PIM2 score was available for 645/744 sepsis survivors and 36/51 non-survivors. Data were log transformed for univariable and multivariable analysis

^d^Data on lactate at pediatric ICU admission were available for 421/744 sepsis survivors and 23/51 non-survivors. Data were log transformed for univariable analysis

^e^Bacterial etiology was confirmed in 428 patients, including 3 patients with mixed invasive pathogens in culture results: these 3 patients have been excluded, leaving 425 patients for analysis

Infection caused by *S*. *pneumoniae* (OR 4.1, 95% CI 1.1–16.0, *P* = 0.04) and illness severity (PIM2 score OR 2.8, 95% CI 1.3–6.1, *P* < 0.01) remained independently significantly associated with mortality in multivariable analysis. A trend towards higher mortality was observed for bacteremia (OR 7.4, 95% CI 1.0–56.6, *P* = 0.06). PICU mortality did not differ significantly across age categories or countries. Also, the presence of an underlying condition at admission to the PICU was not associated with mortality.

The median PICU-free days to day 28 were 23 days (IQR 18–25) and the median hospital length of stay was 12 days (IQR 8–21). PIM2 score (B = − 0.202, *P* < 0.001), invasive *S. pneumoniae* infections (B = − 0.161, *P* = 0.02), and invasive *Staphylococcus aureus* infections (B = − 0.163, *P* = 0.01) were independent predictors of PICU-free days. PIM2 score (B = 0.270, *P* < 0.001), pneumonia (B = 0.145, *P* = 0.04), and invasive *Staphylococcus aureus* infections (B = 0.234, *P* = 0.001) were independent predictors of hospital length of stay (Additional file 1: Table S4).

### Disability

Data on disability at discharge were available on 558/744 survivors (75%). Of these patients, 173/558 (31%) were discharged with disability including 71 patients (13%) with mild overall disability, 39 (7%) with moderate overall disability, 50 (9%) with severe overall disability, 4 (0.7%) who had undergone amputation, 2 (0.4%) with hearing loss, and 7 (1.3%) who had undergone skin graft. Toddlers (34%) and school-aged children (42%) were more often discharged with disability than infants (21%, *P* value <0.05).

Among survivors who did not have an underlying condition at admission to PICU, i.e. previously healthy children, 24% (83/349 patients whose data were available) were discharged with some disability. Disability data were available on 339/421 (81%) survivors of septic shock. In these, 120/339 (35%) patients had disability at PICU discharge, including 45 (13%) with mild overall disability, 29 (9%) with moderate overall disability, 35 (10%) with severe overall disability, 4 (1.2%) who had undergone amputation, and 7 (2.0%) who had undergone skin graft. Outcome as measured by mortality and POPC score was worst in patients admitted with pneumonia (Additional file 1: Figure S3) and in patients with invasive bacterial infections caused by *Streptococcus pneumoniae* (Additional file 1: Figure S4). When comparing patients discharged with and without disability, by univariable and multivariable analysis, the PIM2 score (OR 3.4, 95% CI 1.8–6.4, *P* < 0.001) and infections caused by *Streptococcus pneumoniae* (OR 5.4, 95% CI 1.8–15.8, *P* < 0.01) were independent predictors of disability (Table 3).

**Table 3:** Predictors of disability in survivors of community-acquired sepsis

	No disability at discharge (*n* = 385)	Disability at discharge (*n* = 173)	Univariable odds ratio for disability (95% CI)	*P*	Multivariable odds ratio for disability (95% CI)	*P*
Sex						
Male	196/385 (51%)	90/173 (52%)	Reference			
Female	189/385 (49%)	83/173 (48%)	1.0 (0.7–1.4)	0.81	NA	
Age						
29 days–12 months (infants)	145/385 (38%)	38/173 (22%)	Reference			
1–5 years (toddlers)	135/385 (35%)	70/173 (41%)	2.0 (1.3–3.1)	< 0.01	1.8 (0.8–4.1)	0.14
5–12 years (school-aged children)	55/385 (14%)	39/173 (23%)	2.7 (1.6–4.7)	< 0.001	2.6 (1.0–7.0)	0.05
12–18 years (adolescents)	50/385 (13%)	26/173 (15%)	2.0 (1.1–3.6)	0.02	2.0 (0.7–6.0)	0.20
Time interval from onset of symptoms to hospital admission^a^ (days)	1 (1–3)	1 (1–3)	1.0 (0.9–1.0)	0.16	0.9 (0.9–1.0)	0.07
Immunizations up to date						
No	29/334 (9%)	17/157 (11%)	Reference			
Yes	305/334 (91%)	140/157 (89%)	0.8 (0.4–1.5)	0.45	NA	
Underlying condition						
No	266/385 (69%)	83/173 (48%)	Reference			
Yes	119/385 (31%)	90/173 (52%)	2.4 (1.7–3.5)	< 0.001	1.9 (0.9–3.6)	0.08
Illness severity						
PRISM score	12 (6–19)	16 (11–23)	1.1 (1.0–1.1)	< 0.001	NA	
PIM2 score^b^ (predicted death, %)	3.1 (0.9–7.1)	6.8 (2.3–17.2)	2.7 (1.9–3.9)	< 0.001	3.4 (1.8–6.4)	< 0.001
Lactate at PICU admission^c^ (mmol/L)	1.6 (1.0–3.2)	2.3 (1.2–4.5)	2.0 (1.0–3.8)	0.04	NA	
Invasive ventilation	242/370 (65%)	139/167 (83%)	2.6 (1.7–4.2)	< 0.001	NA	
Inotropes	196/365 (54%)	112/166 (68%)	1.8 (1.2–2.6)	< 0.01	NA	
Bacteremia						
No	262/385 (68%)	112/173 (65%)	Reference			
Yes	123/385 (32%)	61/173 (35%)	1.2 (0.8–1.7)	0.44	NA	
Clinical syndromes						
No focus	146/385 (38%)	48/173 (28%)	0.6 (0.4–0.9)	0.02	0.9 (0.3–2.4)	0.83
Meningitis/encephalitis	74/385 (19%)	49/173 (28%)	1.7 (1.1–2.5)	0.02	1.0 (0.3–3.2)	0.95
Pneumonia	57/385 (15%)	49/173 (28%)	2.3 (1.5–3.5)	< 0.001	1.2 (0.4–3.8)	0.73
Other focus	108/385 (28%)	27/173 (16%)	0.5 (0.3–0.8)	< 0.01	1.0 (0.3–3.0)	0.96
Invasive pathogens						
*N*. *meningitidis*	81/177 (45%)	18/87 (21%)	0.3 (0.2–0.6)	< 0.001	0.5 (0.2–1.3)	0.16
*S*. *pneumoniae*	12/177 (7%)	27/87 (31%)	6.3 (3.0–13.2)	< 0.001	5.4 (1.8–15.8)	< 0.01
Group A streptococcus	31/177 (18%)	15/87 (17%)	1.0 (0.5–2.0)	1.0	NA	
*S*. *aureus*	16/177 (9%)	11/87 (13%)	1.5 (0.7–3.4)	0.34	NA	
Other invasive pathogen	37/177 (21%)	16/87 (18%)	0.9 (0.4–1.6)	0.63	NA	

This study included 795 children with community-acquired sepsis, of whom 173 patients were discharged with disability, i.e. Pediatric Overall Performance Category score 2– 5 [29], need of skin graft, hearing loss, or need of amputation (51 deaths and 186 patients with missing data are not included in this analysis). Multivariable analysis included variables with a *P* value < 0.20 in univariable analysis. Because parameters of illness severity are strongly correlated, only the Pediatric Index of Mortality 2 (PIM2) score [28] has been included in multivariable analysis. Values are reported as counts (percentages) or medians (interquartile ranges), unless stated otherwise

*PRISM* Pediatric Risk of Mortality [27], *NA* not applicable

^a^Time interval from onset of symptoms to hospital admission was available for 372/385 patients without disability and for 154/173 patients with disability at discharge

^b^Data were log transformed for univariable and multivariable analysis

^c^Data on lactate at pediatric ICU admission were available for 247/385 patients without disability and for 109/173 patients with disability at discharge. Data were log transformed for univariable analysis

### Economic impact of vaccine-preventable infections

Assuming an average cost per PICU day of 4000 € and 1000 € per day on a general ward, we estimate an average cost of 42,000 € per vaccine-preventable episode of severe community-acquired infection requiring admission to a PICU. This calculation was based on the mean hospital length of stay (18 days), including mean PICU length of stay (8 days), of the subgroup of patients with vaccine-preventable infections (*n* = 149). Within our consortium, a total of 149 vaccine-preventable cases reflect 43 cases per year. The impact on cost and resource utilization for the hospitals included was estimated at almost 2 million € for potentially vaccine-preventable infections annually.

## Discussion

This prospective multicenter study of 795 children admitted with community-acquired sepsis to European PICUs demonstrates the substantial burden of severe invasive bacterial disease, despite widespread immunization programs, predominantly affecting previously healthy children [30]. Almost one third of survivors (31%) were discharged with disability ranging from mild to severe.

We observed a crude mortality rate of 6% in children admitted with sepsis. Other studies have reported higher mortality of up to 29% in high-income countries, which may relate to the large number of hospital-acquired infections with a disproportionate impact of high-risk patients such as those with oncologic conditions or those undergoing transplant in other cohorts [1, 2, 4, 5, 8, 31–33]. The enrolment criteria in our study were based on the 2005 consensus pediatric sepsis definition, and we included patients with sepsis in addition to patients with severe sepsis and septic shock, which may account for the lower mortality observed. However, most study patients were admitted to the PICU because of single or multiple organ dysfunction, and hence would be expected to meet the Sepsis-3-based sepsis definitions too [21, 34]. The limitations of current pediatric sepsis definitions including the low predictive accuracy of SIRS [35], and the need to adapt Sepsis-3 for pediatric age groups, have been highlighted recently [36].

We observed that 1 out of 3 sepsis survivors were discharged with a disability, including 1 in 10 with severe disability and/or amputation. Notably, 24% of previously healthy children left the hospital with some form of disability. While there is a lack of large studies on pediatric sepsis long-term outcomes, similar incidence of disability has been reported in two other studies, with a decline in functional status observed in 28 to 34% of pediatric sepsis survivors [4, 20]. Others have observed impaired neuropsychological performance and impaired educational functioning [19]. Our findings highlight the need to include disability as an outcome measure in pediatric sepsis trials in the future. More research is required to delineate the nature of the disabilities and to study the add-on effect of sepsis when underlying conditions are already present. Disability in children with underlying conditions could be evaluated more accurately in the future by reporting changes in performance scales between admission and discharge.

Independent risk factors for death, disability, and PICU-free days were illness severity - reflected by severity scores - and invasive pneumococcal infections. Our findings indicate that while current PICU severity scores were calibrated against mortality, PIM performs very well to predict disability as well, which indicates that some patients predicted to die survive, yet with a major impact on functional status. Larger studies are urgently needed to assess long-term impact, as this patient group is at high risk of prolonged dependency on health support, reduced school and work life performance, and reduced quality of life, resulting in an under recognized disproportionate impact of sepsis on our society [14]. Lactate was associated with mortality and the optimal cutoff value of 2.2 mmol/L in serum supports using lactate as a trigger threshold in National Institute for Health and Care Excellence (NICE) UK guidelines [37]. Previous studies have demonstrated the strong association between lactate and mortality, and indicated that both arterial and venous serum lactate level can be used for risk stratification [38–41]. Importantly, our study demonstrated that increased lactate levels at PICU admission were associated with disability too.

An invasive bacterial infection was confirmed in half of the children, which is comparable to pathogen detection rates from 30 to 65% in other studies [1, 4, 5, 33]. The most common community-acquired invasive pathogens in our study were meningococci - especially serogroup B (menB) - and pneumococci - especially serotypes 3 and 10A. Recently, a menB vaccine (Bexsero®) has been licensed for active immunization against menB and this vaccine has been implemented in the Czech Republic and UK routine immunization schedules [42, 43]. It had been anticipated that this vaccine would cover approximately 70 to 80% of MenB strains, depending on geographical region and age [30, 42]. Preliminary data report 93% vaccine uptake of two doses by 12 months of age [44]. Future studies should determine the impact on disease and herd protection. Meningococcal serogroup C immunization resulted in a significant drop in incidence of over 80% in the UK and over 90% in The Netherlands [45–47]. In contrast, invasive MenW disease is increasing and careful monitoring in the coming years is necessary [48]. Immunization against pneumococcal disease is recommended in almost all European countries, and has been proven effective in the decline of invasive pneumococcal infections [49]. In the post-immunization era, the incidence of non-vaccine serotypes has however increased, suggesting serotype replacement [49]. Additionally, vaccine failure does occur. Primary immunodeficiency is present in up to 26% of children > 2 years of age with invasive pneumococcal infections after introduction of vaccination, indicating that infected patients should undergo immunological investigations [50]. Pneumococcal serotype 3 is included in Prevnar 13 (PCV13), but not in Synflorix (PCV10). Limited vaccine effectiveness for serotype 3 has been reported previously [51, 52]. Pathogen detection is complicated by early administration of antibiotics and by low circulating microbial loads [53]. Therefore, new diagnostics to improve pathogen detection and optimal antimicrobial therapy are urgently needed [54].

Despite widespread vaccination campaigns in Europe effectively targeting invasive pneumococcal disease [43, 55–57], the burden due to these potentially vaccine-preventable infections has remained considerable: 17 % of patients with pneumococcal infections have died, while 35% of the patients have been discharged with some form of disability. The pneumococcal mortality rate in our study is slightly higher than mortality rates from other PICU studies [2, 8, 58]. However, those studies relied on ICD-9 codes for organisms and did not evaluate specific culture results. Therefore, a number of infections might have been classified based on non-sterile site cultures (e.g. nasopharyngeal aspirate), and thus could have identified a colonized location, whereas we confirmed each invasive pneumococcal infection by sterile site positive detection. On the other hand, our definition of invasive pneumococcal infection might have skewed the results away from respiratory infections, towards central nervous system infections, as we considered blood, pleural aspirate, and bronchoalveolar lavage as invasive detection sites for pneumonia. These sites are not routinely screened in patients with pneumonia. Nevertheless, our findings emphasize the importance and the need to continuously improve current immunization programs. Additionally, reducing potentially vaccine-preventable infections will have a beneficial effect on economic resources. We estimated an average cost of 42,000 € per vaccine preventable episode of severe community-acquired infection requiring PICU. In comparison, a recent Australian and New Zealand study [5] estimated the mean cost per ICU and ward admission for sepsis and septic shock of AUS$62062 (equal to 39,000 €). Beside direct costs in severe cases requiring PICU admission, the total economic impact of vaccine-preventable diseases encompasses direct costs in cases not requiring PICU admission, and indirect costs related to loss of revenue of caregivers and long-term costs related to permanent disability.

This study has several limitations, most of them relating to the design of this large, international consortium. First, the primary aim of our consortium was to identify genes associated with susceptibility and severity of invasive meningococcal, pneumococcal, staphylococcal, and group A streptococcal infections, which might have caused an enrollment bias in favor of infections caused by these organisms. Therefore, data on the prevalence and distribution of pathogens need to be interpreted with caution. Second, due to the genetic basis of this study, a bias towards enrollment of previously healthy children might have occurred. In our cohort, only 36% of patients had an underlying condition at admission, whereas in other studies percentages varying from 49 to 77% are reported, yet these studies included hospital-acquired infections too [1, 2, 4, 5, 7, 8]. Third, we assessed disability by the easily applicable and well-validated pediatric overall performance scale at discharge [29, 59]. However, there is a fair amount of disability data missing, possibly because of loss to follow up after patients had been transferred back to the local hospital prior to discharge. For patients with disability data available, we did not take further recovery after discharge into account. Fourth, the EUCLIDS consortium represents a network of institutions active in infectious diseases research and was not designed to provide population-based coverage. Hence we are unable to compare findings between countries and we are unable to estimate the impact of vaccine-preventable disease on mortality, morbidity, and costs at population level. Finally, our consortium includes multiple centers from multiple countries, representing a different epidemiological context, healthcare structures, and case-mix. Nevertheless, this study includes the largest prospectively enrolled contemporary cohort of children with community-acquired sepsis in high-income countries. Because previous pediatric sepsis studies included healthcare-associated infections, results from this study especially have implications for policy makers in public health, e.g. to develop immunization strategies. Last, this report differs from previous reports because we included disability as an outcome measure, thereby meeting the need to improve our understanding of the short-term physical effects of sepsis and understanding the implications for sepsis survivors [14].

## Conclusions

This report from high-income countries describes a large cohort of children admitted with community-acquired sepsis to European PICUs, providing contemporary assessment of the epidemiology and characteristics of one of the most common reasons for PICU admission. Our study demonstrates the substantial burden caused by community-acquired sepsis, predominantly affecting previously healthy children. One out of three survivors was discharged with disability, indicating an urgent need for improved recognition, treatment, and follow up of children with sepsis.

## Additional file

Additional file 1: **Figures S1-S4**, **Tables S1-S4**, and the EUCLIDS consortium author list. (DOCX 145 kb)

## Abbreviations

AUROC: Area under the receiver operating characteristic
CI: Confidence interval
EUCLIDS: European Childhood Life-threatening Infectious Disease Study
FP7: 7th Framework Program
HiB: *Haemophilus influenzae* type B
IQR: Interquartile range
MenACWY: Meningococcus serogroups ACWY
MenB: Meningococcus serogroup B
MenC: Meningococcus serogroup C
MenW: Meningococcus serogroup W
NLR: Negative likelihood ratio
NPV: Negative predictive value
OR: Odds ratio
PCV10: Pneumococcal conjugate vaccine 10, Synflorix
PCV13: Pneumococcal conjugate vaccine 13, Prevnar 13
PCV7: Pneumococcal conjugate vaccine 7, Prevnar
PICU: Pediatric Intensive Care Unit
PIM2: Pediatric Index of Mortality
PLR: Positive likelihood ratio
POPC: Pediatric Overall Performance Category
PPSV23: Pneumococcal polysaccharide vaccine 23
PPV: Positive predictive value
PRISM: Pediatric risk of mortality score
SD: Standard deviation
SE: Standard error
SIRS: Systemic inflammatory response syndrome
