# Supplementary material for: Mortality and morbidity in community-acquired sepsis in European pediatric intensive care units: a prospective cohort study from the European Childhood Life-threatening Infectious Disease Study (EUCLIDS)
Source: Crit Care. 2018 May 31;22:143. doi: 10.1186/s13054-018-2052-7 (PMC5984383; doi:10.1186/s13054-018-2052-7)
Supplement: Supplementary file 1 — Figures S1-S4, Tables S1-S4, and the EUCLIDS consortium author list. (DOCX 145 kb) [file 13054_2018_2052_MOESM1_ESM.docx]

**Additional files**

**Mortality and morbidity in community-acquired sepsis in European Pediatric Intensive Care Units: a prospective cohort study from the European Childhood Life-threatening Infectious Disease Study (EUCLIDS)**

Navin P. Boeddha*, Luregn J. Schlapbach*, Gertjan J. Driessen, Jethro A. Herberg, Irene Rivero, Miriam Cebey-López, Daniela S. Klobassa, Ria Philipsen, Ronald de Groot, David P. Inwald, Simon Nadel, Stéphane Paulus, Eleanor Pinnock, Fatou Secka, Suzanne T. Anderson, Rachel S. Agbeko, Christoph Berger, Colin G. Fink, Enitan D. Carrol, Werner Zenz, Michael Levin, Michiel van der Flier, Federico Martinón-Torres, Jan A. Hazelzet^, Marieke Emonts^, on behalf of the EUCLIDS consortium

*^ These authors have contributed equally to the work presented in this manuscript.

**CONTENT**

**Figure S1:** clinical presentations (n=795) within age categories.

**Figure S2:** AUROC curve analysis for lactate as predictor for mortality.

Sensitivity, specificity, PPV, NPV, PLR, and NLR for 2.2 mmol/L as optimal cut-off value of lactate.

**Figure S3:** unadjusted mortality and POPC score of patients with meningitis/encephalitis, pneumonia, and no focus.

**Figure S4:** unadjusted mortality and POPC score of patients with invasive meningococcal, pneumococcal, Group A streptococcal, and S. aureus infections.

**Table S1:** routine immunization schedules and uptake.

**Table S2:** the Pediatric Overall Performance Category (POPC) scale.

**Table S3:** characteristics of *Haemophilus influenza*, meningococcal, and pneumococcal sepsis, including serotypes/serogroups and clinical presentation of deaths and patients with disability.

**Table S4:** predictors of PICU-free days and hospital length of stay in children with community-acquired sepsis.

**Consortium:** EUCLIDS consortium author list

## Figure S1: clinical presentations (n=795) within age categories.

A) Clinical presentations of patients admitted to PICU for community-acquired sepsis. Most common were patients in whom a focus of infection could not be identified (n=278, 35%), meningitis/encephalitis (n=182, 23%) and pneumonia (n=149, 19%).

B) The percentages of clinical presentations within four age categories were similar, apart from Osteomyelitis/septic arthritis. **P*-value<0.05.

## Figure S2: AUROC curve analysis for lactate as predictor for mortality. Sensitivity, specificity, PPV, NPV, PLR, and NLR for 2.2 mmol/L as optimal cut-off value of lactate.


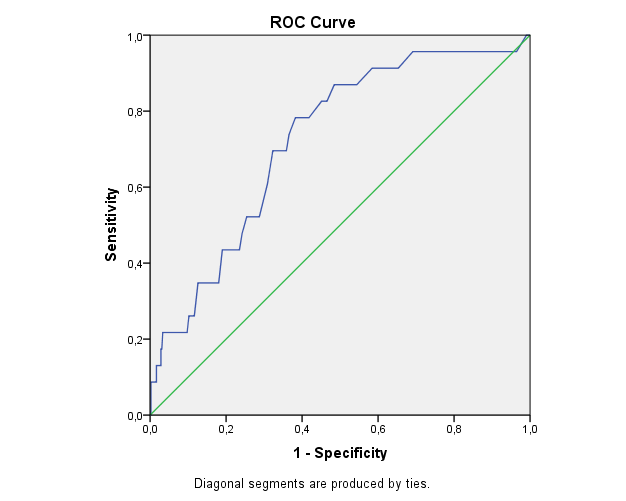


|  | **Death** | **No death** | **Total** |
| --- | --- | --- | --- |
| **Lactate ≥ 2.2 mmol/L** | 18 | 169 | 187 |
| **Lactate < 2.2 mmol/L** | 5 | 252 | 257 |
| Total | 23 | 421 | 444 |

Sensitivity: 18/23 = 78%

Specificity: 252/421 = 60%

PPV: 18/187 = 10%

NPV: 252/257 = 98%

PLR: (18/23)/(169/421) = 1.9

NLR: (5/23)/(252/421) = 0.4

## Figure S3: unadjusted mortality and POPC score of patients with meningitis/encephalitis, pneumonia, and no focus.

Patients admitted with pneumonia (n=149) had a crude mortality of 7% (n=11), while an additional 33% (n=49) were discharged with mild, moderate or severe disability.

## Figure S4: unadjusted mortality and POPC score of patients with invasive meningococcal, pneumococcal, Group A streptococcal, and S. aureus infections.

Invasive bacterial infections caused by *Streptococcus pneumoniae* (n=78) resulted in the worst crude outcome; 17% mortality (n=13), while an additional 33% (n=26) were discharged with POPC scores mild, moderate or severe disability.

## Table S1: Immunization schedules and uptake

|  | **Austria^1^** | **Germany^1^** | **Lithuania^1^** | **Spain^2^** | **Switzerland^3^** | **The**  **Netherlands^4^** | **United Kingdom^5,6^** |
| --- | --- | --- | --- | --- | --- | --- | --- |
| **HiB**  Introduced  Uptake | 1992 | 1990 | 2004 | 2000  96% | 1990  89% | 1993  95% | 1992  94% |
| **MenACWY**  Introduced  Uptake | 2013 | - | - | - | - | - | 2015  84% |
| **MenB**  Introduced  Uptake | 2015 | - | 2018 | - | - | - | 2015  93% |
| **MenC**  Introduced  Uptake | 2009 | 2006 | - | 2002  98% | 2006  72% | 2002  95% | 1999  92% |
| **PCV7**  Introduced  Uptake | 2004 | 2006 | - | 2006 | 2006 | 2006 | 2006 |
| **PCV10**  Introduced  Uptake | 2011 | 2009 | 2014 | - | - | 2011  94% | 2009 |
| **PCV13**  Introduced  Uptake | - | 2009 | - | 2012 | 2011  80% | - | 2010  94% |
| **PPSV23**  Introduced  Uptake | - | - | - | - |  | - |  |

For immunizations where uptake is not reported, data are unavailable or unknown.

References:

1. Personal communication from pediatric infectious diseases specialists in respective country

2. Ministerio de Sanidad, Servicios Sociales e Igualdad. https://www.msssi.gob.es/profesionales/saludPublica/prevPromocion/vacunaciones/docs/CoberturasVacunacion/Todas_las_tablas.pdf

3. [Bundesamt für Gesundheit BAG](https://www.bag.admin.ch/). https://www.bag.admin.ch/bag/de/home/themen/mensch-gesundheit/uebertragbare-krankheiten/impfungen-prophylaxe/informationen-fachleute-gesundheitspersonal.html

4. E.A. van Lier et al. Vaccinatiegraad Rijksvaccinatieprogramma Nederland. Verslagjaar 2016.

5. Public Health England, Health Protection Report, 27 January 2018. Vaccine coverage estimates for the school based meningococcal ACWY (MenACWY) adolescent vaccination programme in England, to 31 August 2017 and Preliminary vaccine coverage estimates for the meningococcal B (MenB) immunisation programme for England, update from August to December 2017.

6. NHS digital, Childhood Vaccination Coverage Statistics: http://bit.ly/child_vaccstats_annual

## Table S2: The Pediatric Overall Performance Category (POPC) scale.

| **Score** | **Category** | **Description** |
| --- | --- | --- |
| **1** | Good overall performance | Normal; at age-appropriate level; school-age child attending regular school classroom.  Healthy, alert, and capable of normal activities of daily life. |
| **2** | Mild overall disability | Conscious; alert, and able to interact at age-appropriate level; school-age child attending regular school classroom but grade perhaps not appropriate for age; possibility of mild neurologic deficit.  Possibility of minor physical problem that is still compatible with normal life; conscious and able to function independently. |
| **3** | Moderate overall disability | Conscious; sufficient cerebral function for age-appropriate independent activities of daily life; school-age child attending special education classroom and/or learning deficit present  Possibility of moderate disability from noncerebral systems dysfunction alone or with cerebral system dysfunction; conscious and performs independent activities of daily life but is disabled for competitive performance in school. |
| **4** | Severe overall disability | Conscious; dependent on others for daily support because of impaired brain function.  Possibility of severe disability from noncerebral systems dysfunction alone or with cerebral system dysfunction; conscious but dependent on others for activities of daily living support. |
| **5** | Coma or vegetative state | Any degree of coma without the presence of all brain death criteria; unawareness, even if awake in appearance, without interaction with environment; cerebral unresponsiveness and no evidence of cortex function (not aroused by verbal stimuli); possibility of some reflexive response, spontaneous eye-opening, and sleep-wake cycles. |
| **6** | Brain death | Apnea, areflexia, and/or electroencephalographic silence. |

## Table S3: characteristics of *Haemophilus influenza*, meningococcal, and pneumococcal sepsis, including serotypes/serogroups and clinical presentation of deaths and patients with disability.

|  | **H. influenza (n=12)** | **Meningococcal (n=131)** | **Pneumococcal sepsis (n=78)** |
| --- | --- | --- | --- |
| **Sex** (male n, %) | 7 (58%) | 73 (56%) | 43 (55%) |
| **Age** | 12m [10m-3y] | 20m [8m-5y] | 21m [8m-6y] |
| **Country**  Austria  Germany  Lithuania  Spain  Switzerland  The Netherlands  United Kingdom | -  -  -  1 (8%)  8 (67%)  1 (8%)  2 (17%) | 15 (12%)  6 (5%)  10 (8%)  32 (24%)  16 (12%)  9 (7%)  43 (33%) | 1 (1%)  1 (1%)  -  6 (8%)  31 (40%)  21 (27%)  18 (23%) |
| **Serotype/-group** (n, %) | B (10, 83%)  Non-B (1, 8%)  Unspecified (1, 8%) | B (89, 68%)  C (9, 7%)  W135 (8, 6%)  Y (1, 1%)  Unspecified (24, 18%) | ***7-valent:***  6B (1, 1%)  ***10-valent:***  1 (2, 3%)  7F (2, 3%)  ***13-valent:***  3 (7, 9%)  19A (4, 5%)  ***23-valent:***  8 (2, 3%) 15B (1, 1%),  10A (6, 8%) 17F (1, 1%)  11A (2, 3%) 22F (2, 3%)  12F (1, 1%) 33F (1, 1%)  ***Other:***  12 (1, 1%) 23 (1, 1%) 24F (1, 1%)  15C (2, 3%) 23A (1, 1%) 27 (1, 1%)  17 (1, 1%) 23B (1, 1%) 33 (1, 1%)  21 (1, 1%) 24 (4, 5%) 38 (1, 1%)  15B or 15C unspecified (3, 4%)  Unknown (27, 35%) |
| **Immunization status of potentially VPI**^*^ (n, %)  Complete immunization  Incomplete immunization - age  Incomplete immunization - avail.  No immunization  Unknown | 10 (83%)  2 (20%)  5 (50%)  0 (0%)  1 (10%)  2 (20%) | 107 (82%)  1 (1%)  5 (5%)  82 (77%)  2 (2%)  17 (16%) | 32 (41%)  0 (0%)  1 (3%)  25 (78%)  0 (0%)  6 (19%) |
| **Clinical presentation**  No focus  Meningitis/encephalitis  Pneumonia  Other | 1 (8%)  4 (33%)  4 (33%)  3 (25%) | 68 (52%)  61 (47%)  1 (1%)  1 (1%) | 5 (6%)  51 (66%)  21 (27%)  1 (1%) |
| **Deaths sub-analysis**  Serotype/-group (n, %)  Clinical presentation (n, %) | **0 (0%)**  -  - | **11 (8%)**  B (7, 64%)  C (2, 18%),  Unspecified (2, 18%)  No focus (10, 91%)  Meningitis/enceph (1, 9%) | **13 (17%)**  3 (1, 8%) 15C (2, 15%) 23A (1, 8%)  8 (1, 8%) 21 (1, 8%) 24 (2, 15%)  12F (1, 8%) 22F (1, 8%) unkn. (3, 23%)    No focus (2, 15%)  Meningitis/encephalitis (7, 54%)  Pneumonia (3, 23%)  Other (1, 8%) |
| **Disability sub-analysis**  Serotype/-group (n, %)  Clinical presentation (n, %) | **0 (0%)**  -  - | **18 (14%)**  B (11, 61%)  W135 (3, 17%),  Unspecified (4, 22%)  No focus (12, 67%)  Meningitis/enceph (6, 33%) | **27 (35%)**  6B (1, 4%) 11A (1, 4%) 17F (1, 4%)  7F (1, 4%) 12 (1, 4%) 19A (1, 4%)  8 (1, 4%) 15B (1, 4%) 27 (1, 4%)  10A (3, 11%) 17 (1, 4%) 33 (1, 4%)  15B or 15C unspecified (1, 4%)  Unknown (12, 44%)  Meningitis/encephalitis (25, 93%)  Pneumonia (2, 7%) |

Values are reported as counts (percentages) or medians [interquartile ranges], unless stated otherwise. VPI = vaccine preventable infections.

^*^Immunization: complete = immunizations up-to-date according to local schedule and sufficient doses for protection received; incomplete age = immunizations up-to-date according to local schedule, but sufficient doses for protection not received because of young age; incomplete - availability = immunizations up-to-date according to local schedule, but immunization not received because of unavailability in local schedule; no = immunizations not up-to-date according to local schedule, and therefore doses not received.

## Table S4: predictors of PICU-free days and hospital length of stay in children with community-acquired sepsis.

|  | **PICU-FREE DAYS** | | | | **HOSPITAL LENGTH OF STAY** | | | |
| --- | --- | --- | --- | --- | --- | --- | --- | --- |
|  | **Univariable analysis**  **Standardized B** | ***P*** | **Multivariable analysis Standardized B** | ***P*** | **Univariable analysis Standardized B** | ***P*** | **Multivariable analysis**  **Standardized B** | ***P*** |
| **Sex** (Female) | -0.015 | 0.67 | .. |  | -0.035 | 0.50 | .. |  |
| **Age**  29d-12m (infants)  1-5y (toddlers)  5-12y (school aged children)  12-18y (adolescents) | 0.030  0.020  -0.030  -0.035 | 0.41  0.58  0.40  0.33 | ..  ..  ..  .. |  | -0.034  -0.004  0.054  -0.006 | 0.51  0.93  0.30  0.91 | ..  ..  ..  .. |  |
| **Time onset symptoms to hospital admission^*^** (days) | -0.071 | 0.08 | -0.060 | 0.30 | 0.061 | 0.25 | .. |  |
| **Immunizations up-to-date** | -0.004 | 0.92 | .. |  | 0.012 | 0.83 | .. |  |
| **Underlying condition** | -0.183 | <0.001 | -0.067 | 0.25 | 0.085 | 0.10 | 0.062 | 0.36 |
| **Illness severity**  PRISM score  PIM2 score^~^ (predicted death, %)  Lactate PICU admission^@^ (mmol/L)  Invasive ventilation  Inotropes | -0.326  -0.244  -0.265  -0.338  -0.331 | <0.001  <0.001  <0.001  <0.001  <0.001 | ..  -0.202  ..  ..  .. | 0.001 | 0.303  0.226  0.179  0.245  0.147 | <0.001  <0.001  <0.01  <0.001  <0.01 | ..  0.268  ..  ..  .. | <0.001 |
| **Bacteremia** | -0.066 | 0.07 | -0.061 | 0.32 | 0.094 | 0.07 | 0.107 | 0.13 |
| **Clinical syndromes**  No focus  Meningitis/encephalitis  Pneumonia  Other focus | 0.019  0.104  -0.197  0.058 | 0.60  <0.01  <0.001  0.11 | ..  0.109  -0.129  0.030 | 0.12  0.06  0.67 | -0.083  -0.052  0.138  0.027 | 0.11  0.31  <0.01  0.60 | -0.060  ..  0.148  .. | 0.43  0.04 |
| **Invasive pathogens**  N. meningitidis  S. pneumoniae  Group A streptococcus  S. aureus  Other pathogen | 0.163  -0.144  -0.078  -0.096  0.080 | 0.001  <0.01  0.11  0.05  0.11 | 0.025  -0.161  -0.020  -0.163  -0.018 | 0.79  0.02  0.80  0.01  0.81 | -0.195  -0.015  0.103  0.239  -0.024 | <0.01  0.84  0.15  0.001  0.74 | -0.012  ..  0.072  0.249  .. | 0.89  0.37  0.001 |
| ***R*^2^** |  |  | 0.17 |  |  |  | 0.20 |  |
| **ANOVA: *df, F*** |  |  | 11, 4.920 | <0.001 |  |  | 8, 5.664 | <0.001 |

## EUCLIDS consortium author list

### EUCLIDS consortium (www.euclids-project.eu) is composed by:

### Imperial College London (UK)

Principal and co-investigators:

Michael Levin (grant application, EUCLIDS Coordinator)

Lachlan Coin (bioinformatics)

Stuart Gormley (clinical coordination)

Shea Hamilton (proteomics)

Jethro Herberg (grant application, PI)

Bernardo Hourmat (project management)

Clive Hoggart (statistical genomics)

Myrsini Kaforou (bioinformatics)

Vanessa Sancho-Shimizu (genetics)

Victoria Wright (grant application, scientific coordination)

Consortium members at Imperial College:

Amina Abdulla

Paul Agapow

Maeve Bartlett

Evangelos Bellos

Hariklia Eleftherohorinou

Rachel Galassini

David Inwald

Meg Mashbat

Stefanie Menikou

Sobia Mustafa

Simon Nadel

Rahmeen Rahman

Clare Thakker

### EUCLIDS UK Clinical Network

Poole Hospital NHS Foundation Trust, Poole: Dr S Bokhandi (PI), Sue Power, Heather Barham

Cambridge University Hospitals NHS Trust, Cambridge: Dr N Pathan (PI), Jenna Ridout, Deborah White, Sarah Thurston

University Hospital Southampton, Southampton: Prof S Faust (PI), Dr S Patel (co-investigator), Jenni McCorkell.

Nottingham University Hospital NHS Trust: Dr P Davies (PI), Lindsey Crate, Helen Navarra, Stephanie Carter

University Hospitals of Leicester NHS Trust, Leicester: Dr R Ramaiah (PI), Rekha Patel

Portsmouth Hospitals NHS Trust, London: Dr Catherine Tuffrey (PI), Andrew Gribbin, Sharon McCready

Great Ormond Street Hospital, London: Dr Mark Peters (PI), Katie Hardy, Fran Standing, Lauren O’Neill, Eugenia Abelake

King’s College Hospital NHS Foundation Trust, London; Dr Akash Deep (PI), Eniola Nsirim

Oxford University Hospitals NHS Foundation Trust, Oxford Prof A Pollard (PI), Louise Willis, Zoe Young

Kettering General Hospital NHS Foundation Trust, Kettering: Dr C Royad (PI), Sonia White

Central Manchester NHS Trust, Manchester: Dr PM Fortune (PI), Phil Hudnott

**SERGAS (Spain)**

Principal Investigators:

Federico Martinón-Torres^1^

Antonio Salas^1,2^

GENVIP RESEARCH GROUP (in alphabetical order):

Fernando Álvez González^1^, Ruth Barral-Arca^1,2^, Miriam Cebey-López^1^, María José Curras-Tuala^1,2^, Natalia García^1^, Luisa García Vicente^1^, Alberto Gómez-Carballa^1,2^, Jose Gómez Rial^1^, Andrea Grela Beiroa^1^, Antonio Justicia Grande^1^, Pilar Leboráns Iglesias^1^, Alba Elena Martínez Santos^1^, Federico Martinón-Torres^1^, Nazareth Martinón-Torres^1^, José María Martinón Sánchez^1^, Beatriz Morillo Gutiérrez^1^, Belén Mosquera Pérez^1^, Pablo Obando Pacheco^1^, Jacobo Pardo-Seco^1,2^, Sara Pischedda^1,2^, Irene Rivero Calle^1^, Carmen Rodríguez-Tenreiro^1^, Lorenzo Redondo-Collazo^1^, Antonio Salas Ellacuriaga^1,2^, Sonia Serén Fernández^1^, María del Sol Porto Silva^1^, Ana Vega^1,3,^ Lucía Vilanova Trillo^1^.

^1^ Translational Pediatrics and Infectious Diseases, Pediatrics Department, Hospital Clínico Universitario de Santiago, Santiago de Compostela, Spain, and GENVIP Research Group (www.genvip.org), Instituto de Investigación Sanitaria de Santiago, Galicia, Spain.

^2^ Unidade de Xenética, Departamento de Anatomía Patolóxica e Ciencias Forenses, Instituto de Ciencias Forenses, Facultade de Medicina, Universidade de Santiago de Compostela, and GenPop Research Group, Instituto de Investigaciones Sanitarias (IDIS), Hospital Clínico Universitario de Santiago, Galicia, Spain

^3^ Fundación Pública Galega de Medicina Xenómica, Servizo Galego de Saúde (SERGAS), Instituto de Investigaciones Sanitarias (IDIS), and Grupo de Medicina Xenómica, Centro de Investigación Biomédica en Red de Enfermedades Raras (CIBERER), Universidade de Santiago de Compostela (USC), Santiago de Compostela, Spain

EUCLIDS SPANISH CLINICAL NETWORK:

Susana Beatriz Reyes^1^, María Cruz León León^1^, Álvaro Navarro Mingorance^1^, Xavier Gabaldó Barrios^1^, Eider Oñate Vergara^2^, Andrés Concha Torre^3^, Ana Vivanco^3^, Reyes Fernández^3^, Francisco Giménez Sánchez^4^, Miguel Sánchez Forte^4^, Pablo Rojo^5^, J.Ruiz Contreras^5^, Alba Palacios^5^, Cristina Epalza Ibarrondo^5^, Elizabeth Fernández Cooke^5^, Marisa Navarro^6^, Cristina Álvarez Álvarez^6^, María José Lozano^6^, Eduardo Carreras^7^, Sonia Brió Sanagustín^7^, Olaf Neth^8^, Mª del Carmen Martínez Padilla^9^, Luis Manuel Prieto Tato^10^, Sara Guillén^10^, Laura Fernández Silveira^11^, David Moreno^12^.

^1^ Hospital Clínico Universitario Virgen de la Arrixaca; Murcia, Spain.

^2^ Hospital de Donostia; San Sebastián, Spain.

^3^ Hospital Universitario Central de Asturias; Asturias, Spain.

^4^ Complejo Hospitalario Torrecárdenas; Almería, Spain.

^5^ Hospital Universitario 12 de Octubre; Madrid, Spain.

^6^ Hospital General Universitario Gregorio Marañón; Madrid, Spain.

^7^ Hospital de la Santa Creu i Sant Pau; Barcelona, Spain.

^8^ Hospital Universitario Virgen del Rocío; Sevilla, Spain.

^9^ Complejo Hospitalario de Jaén; Jaén, Spain.

^10^ Hospital Universitario de Getafe; Madrid, Spain.

^11^ Hospital Universitario y Politécnico de La Fe; Valencia, Spain.

^12^ Hospital Regional Universitario Carlos Haya; Málaga, Spain.

**Pediatric Dutch Bacterial Infection Genetics (PeD-BIG) network (The Netherlands)**

Steering committee:

**Coordination:** R. de Groot ^1^, A.M. Tutu van Furth ^2^, M. van der Flier ^1^

**Coordination Intensive Care**: N.P. Boeddha ^3^, G.J.A. Driessen ^3^, M. Emonts ^3, 4, 5^, J.A. Hazelzet ^3^

**Other members**: T.W. Kuijpers ^7^, D. Pajkrt ^7^, E.A.M. Sanders ^6^ , D. van de Beek ^8^, A. van der Ende ^8^

**Trial coordinator**: H.L.A. Philipsen ^1^

Local investigators (in alphabetical order):

A.O.A. Adeel ^9^, M.A. Breukels ^10^, D.M.C. Brinkman ^11^, C.C.M.M. de Korte ^12^, E. de Vries ^13^, W.J. de Waal ^15^, R. Dekkers ^15^, A. Dings-Lammertink ^16^, R.A. Doedens ^17^, A.E. Donker ^18^, M. Dousma^19^, T.E. Faber ^20^, G.P.J.M. Gerrits^21^, J.A.M. Gerver ^22^, J. Heidema ^23^, J. Homan-van der Veen ^24^, M.A.M. Jacobs ^25^, N.J.G. Jansen ^6^, P. Kawczynski ^26^, K. Klucovska ^27^, M.C.J. Kneyber ^28^, Y. Koopman-Keemink ^29^, V.J. Langenhorst ^30^, J. Leusink ^31^, B.F. Loza ^32^, I.T. Merth ^33^, C.J. Miedema ^34^, C. Neeleman ^1^, J.G. Noordzij ^35^, C.C. Obihara ^36^, A.L.T. van Overbeek – van Gils ^37^, G.H. Poortman ^38^,S.T. Potgieter ^39^, J. Potjewijd ^40^, P.P.R. Rosias ^41^, T. Sprong ^21^, G.W. ten Tussher ^42^, B.J. Thio ^43^, G.A. Tramper-Stranders ^44^, M. van Deuren ^1^, H. van der Meer ^2^, A.J.M. van Kuppevelt ^45^, A.M. van Wermeskerken ^46^, W.A. Verwijs ^47^, T.F.W. Wolfs ^4^.

1. Radboud University Medical Center – Amalia Children’s Hospital, Nijmegen, The Netherlands
2. Vrije Universiteit University Medical Center, Amsterdam, The Netherlands
3. Erasmus Medical Center – Sophia Children’s Hospital, Rotterdam, The Netherlands
4. Institute of Cellular Medicine, Newcastle University, Newcastle upon Tyne, United Kingdom
5. Paediatric Infectious Diseases and Immunology Department, Newcastle upon Tyne Hospitals Foundation Trust, Great North Children's Hospital, Newcastle upon Tyne, United Kingdom
6. University Medical Center Utrecht – Wilhelmina Children’s Hospital, Utrecht, The Netherlands
7. Academic Medical Center – Emma Children’s Hospital, University of Amsterdam, Amsterdam, The Netherlands
8. Academic Medical Center, University of Amsterdam, Amsterdam, The Netherlands
9. Kennemer Gasthuis, Haarlem, The Netherlands
10. Elkerliek Hospital, Helmond, The Netherlands
11. Alrijne Hospital, Leiderdorp, The Netherlands
12. Beatrix Hospital, Gorinchem, The Netherlands
13. Jeroen Bosch Hospital, ‘s-Hertogenbosch, The Netherlands
14. Diakonessenhuis, Utrecht, The Netherlands
15. Maasziekenhuis Pantein, Boxmeer, The Netherlands
16. Gelre Hospitals, Zutphen, The Netherlands
17. Martini Hospital, Groningen, The Netherlands
18. Maxima Medical Center, Veldhoven, The Netherlands
19. Gemini Hospital, Den Helder, The Netherlands
20. Medical Center Leeuwarden, Leeuwarden, The Netherlands
21. Canisius-Wilhelmina Hospital, Nijmegen, The Netherlands
22. Rode Kruis Hospital, Beverwijk, The Netherlands
23. St. Antonius Hospital, Nieuwegein, The Netherlands
24. Deventer Hospital, Deventer, The Netherlands
25. Slingeland Hospital, Doetinchem, The Netherlands
26. Refaja Hospital, Stadskanaal, The Netherlands
27. Bethesda Hospital, Hoogeveen, The Netherlands
28. University Medical Center Groningen, Beatrix Children’s hospital, Groningen, The Netherlands
29. Haga Hospital – Juliana Children’s Hospital, Den Haag, The Netherlands
30. Isala Hospital, Zwolle, The Netherlands
31. Bernhoven Hospital, Uden, The Netherlands
32. VieCuri Medical Center, Venlo, The Netherlands
33. Ziekenhuisgroep Twente, Almelo-Hengelo, The Netherlands
34. Catharina Hospital, Eindhoven, The Netherlands
35. Reinier de Graaf Gasthuis, Delft, The Netherlands
36. ETZ Elisabeth, Tilburg, The Netherlands
37. Scheper Hospital, Emmen, The Netherlands
38. St. Jansdal Hospital, Hardewijk, The Netherlands
39. Laurentius Hospital, Roermond, The Netherlands
40. Isala Diaconessenhuis, Meppel, The Netherlands
41. Zuyderland Medical Center, Sittard-Geleen, The Netherlands
42. Westfriesgasthuis, Hoorn, The Netherlands
43. Medisch Spectrum Twente, Enschede, The Netherlands
44. St. Franciscus Gasthuis, Rotterdam, The Netherlands
45. Streekziekenhuis Koningin Beatrix, Winterswijk, The Netherlands
46. Flevo Hospital, Almere, The Netherlands
47. Zuwe Hofpoort Hospital, Woerden, The Netherlands

**Swiss Pediatric Sepsis Study (Switzerland)**

***Steering Committee****: Luregn J Schlapbach, MD, FCICM ^1,2,3^, Philipp Agyeman, MD ^1^, Christoph Aebi, MD ^1^, Christoph Berger, MD ^1^*

Luregn J Schlapbach, MD, FCICM ^1,2,3^, Philipp Agyeman, MD ^1^, Christoph Aebi, MD ^1^, Eric Giannoni, MD ^4,5^, Martin Stocker, MD ^6^, Klara M Posfay-Barbe, MD ^7^, Ulrich Heininger, MD ^8^, Sara Bernhard-Stirnemann, MD ^9^, Anita Niederer-Loher, MD ^10^, Christian Kahlert, MD ^10^, Paul Hasters, MD ^11^, Christa Relly, MD ^12^, Walter Baer, MD ^13^, Christoph Berger, MD ^12^ **for the Swiss Pediatric Sepsis Study**

^1.^ Department of Pediatrics, Inselspital, Bern University Hospital, University of Bern, Switzerland

^2.^ Paediatric Critical Care Research Group, Mater Research Institute, University of Queensland, Brisbane, Australia

^3.^ Paediatric Intensive Care Unit, Lady Cilento Children’s Hospital, Children’s Health Queensland, Brisbane, Australia

^4.^ Service of Neonatology, Lausanne University Hospital, Lausanne, Switzerland

^5.^ Infectious Diseases Service, Lausanne University Hospital, Lausanne, Switzerland

^6.^ Department of Pediatrics, Children’s Hospital Lucerne, Lucerne, Switzerland

^7.^ Pediatric Infectious Diseases Unit, Children’s Hospital of Geneva, University Hospitals of Geneva, Geneva, Switzerland

^8.^ Infectious Diseases and Vaccinology, University of Basel Children’s Hospital, Basel, Switzerland

^9.^ Children’s Hospital Aarau, Aarau, Switzerland

^10.^ Division of Infectious Diseases and Hospital Epidemiology, Children’s Hospital of Eastern Switzerland St. Gallen, St. Gallen, Switzerland

^11.^ Department of Neonatology, University Hospital Zurich, Zurich, Switzerland

^12.^ Division of Infectious Diseases and Hospital Epidemiology, and Children’s Research Center, University Children’s Hospital Zurich, Switzerland

^13.^ Children’s Hospital Chur, Chur, Switzerland

**Liverpool partner (UK)**

Principal Investigators :

Enitan Carrol^1^

Stéphane Paulus ^1,2^

ALDER HEY SERIOUS PAEDIATRIC INFECTION RESEARCH GROUP (ASPIRE) (in alphabetical order):

Hannah Frederick^3^, Rebecca Jennings^3^ , Joanne Johnston^3^, Rhian Kenwright^3^

^1^ Department of Clinical Infection, Microbiology and Immunology, University of Liverpool Institute of Infection and Global Health , Liverpool, England

^2^ Alder Hey Children’s Hospital, Department of Infectious Diseases, Eaton Road, Liverpool, L12 2AP

^3^ Alder Hey Children’s Hospital, Clinical Research Business Unit, Eaton Road, Liverpool, L12 2AP

**Micropathology Ltd (UK)**

Colin G Fink^1,2^, Elli Pinnock^1^

^1^Micropathology Ltd Research and Diagnosis

^2^University of Warwick

**Newcastle partner (UK)**

Principle Investigator:

Marieke Emonts^1,2^

Co-Investigator:

Rachel Agbeko^1,3^

^1^ Institute of Cellular Medicine, Newcastle University, Newcastle upon Tyne, United Kingdom

^2^ Paediatric Infectious Diseases and Immunology Department, Newcastle upon Tyne Hospitals Foundation Trust, Great North Children's Hospital, Newcastle upon Tyne, United Kingdom

^3^ Paediatric Intensive Care Unit, Newcastle upon Tyne Hospitals Foundation Trust, Great North Children's Hospital, Newcastle upon Tyne, United Kingdom

**Gambia partner (The Gambia)**

Suzanne Anderson: Principal Investigator and West African study oversight

Fatou Secka: Clinical research fellow and study co-ordinator

Additional Gambia site team (consortium members):

Kalifa Bojang: co-PI

Isatou Sarr: Senior laboratory technician

Ngane Kebbeh: Junior laboratory technician

Gibbi Sey: lead research nurse Medical Research Council Clinic

Momodou Saidykhan: lead research nurse Edward Francis Small Teaching Hospital

Fatoumatta Cole: Data manager

Gilleh Thomas: Data manager

Martin Antonio: Local collaborator

Medical Research Council Unit Gambia

PO Box 273

Banjul

The Gambia

**Austrian partner**

**PI:** Werner Zenz^1^

**Co-Investigators/Steering committee:**

Daniela S. Klobassa^1^, Alexander Binder^1^, Nina A. Schweintzger^1^, Manfred Sagmeister^1^

^1^University Clinic of Paediatrics and Adolescent Medicine, Department of General Paediatrics, Medical University Graz, Austria

**Austrian network, participating centres in Austria, Germany, Italy, Serbia, Lithuania, patient recruitment (in alphabetical order):**

Hinrich Baumgart^1^, Markus Baumgartner^2^, Uta Behrends^3^, Ariane Biebl^4^, Robert Birnbacher^5^, Jan-Gerd Blanke^6^, Carsten Boelke^7^, Kai Breuling^3^, Jürgen Brunner^8^, Maria Buller^9^, Peter Dahlem^10^, Beate Dietrich^11^, Ernst Eber^12^, Johannes Elias^13^, Josef Emhofer^2^, Rosa Etschmaier^14^, Sebastian Farr^15^, Ylenia Girtler^16^, Irina Grigorow^17^, Konrad Heimann^18^, Ulrike Ihm^19^, Zdenek Jaros^20^, Hermann Kalhoff^21^, Wilhelm Kaulfersch^22^, Christoph Kemen^23^, Nina Klocker^24^, Bernhard Köster^25^, Benno Kohlmaier^26^, Eleni Komini^27^, Lydia Kramer^3^, Antje Neubert^28^, Daniel Ortner^29^, Lydia Pescollderungg^16^, Klaus Pfurtscheller^30^, Karl Reiter^31^, Goran Ristic^32^, Siegfried Rödl^30^, Andrea Sellner^26^, Astrid Sonnleitner^26^, Matthias Sperl^33^, Wolfgang Stelzl^34^, Holger Till^1^, Andreas Trobisch^26^, Anne Vierzig^35^, Ulrich Vogel^12^, Christina Weingarten^36^, Stefanie Welke^37^, Andreas Wimmer^38^, Uwe Wintergerst^39^, Daniel Wüller^40^, Andrew Zaunschirm^41^, Ieva Ziuraite^42^, Veslava Žukovskaja^42^

^1^Department of Pediatric and Adolescence Surgery, Division of General Pediatric Surgery, Medical University Graz, Austria

^2^Department of Pediatrics, General Hospital of Steyr, Austria

^3^Department of Pediatrics/Department of Pediatric Surgery, Technische Universität München (TUM), Munich, Germany

^4^Department of Pediatrics, Kepler University Clinic, Medical Faculty of the Johannes Kepler University, Linz, Austria

^5^Department of Pediatrics and Adolesecent Medicine LKH Villach, Austria

^6^Department of Pediatrics and Adolescent Medicine and Neonatology, Hospital Ludmillenstift, Meppen, Germany

^7^Hospital for Children's and Youth Medicine, Oberschwabenklinik, Ravensburg, Germany

^8^Department of Pediatrics, Medical University Innsbruck, Austria

^9^Clinic for Paediatrics and Adolescents Medicine, Sana Hanse-Klinikum Wismar, Germany

^10^Departement of Pediatrics, Medical Center Coburg, Germany

^11^University Medicine Rostock, Department of Pediatrics (UKJ), Rostock, Germany

^12^Department of Pulmonology, Medical University Graz, Austria

^13^Institute for Hygiene and Microbiology, University of Würzburg, Germany

^14^Clinical Institute of Medical and Chemical Laboratory Diagnostics, Medical University Graz, Austria

^15^Department of Pediatric Orthopedics and Adult Foot and Ankle Surgery, Orthopedic Hospital Speising, Vienna, Austria

^16^Department of Paediatrics, Regional Hospital Bolzano, Italy

^17^Department of Pediatrics and Adolescent Medicine, General Hospital Hochsteiermark/Leoben, Austria

^18^Department of Neonatology and Paediatric Intensive Care, Children's University Hospital, RWTH Aachen, Germany

^19^Paediatric Intensive Care Unit, Department of Paediatric Surgery, Donauspital Vienna, Austria

^20^Department of Pediatrics, General Public Hospital, Zwettl, Austria

^21^Pediatric Clinic Dortmund, Germany

^22^Department of Pediatrics and Adolescent Medicine, Klinikum Klagenfurt am Wörthersee, Klagenfurt, Austria

^23^Catholic Children's Hospital Wilhelmstift, Department of Pediatrics, Hamburg, Germany

^24^Department of Pediatrics, Krankenhaus Dornbirn, Austria

^25^Children’s Hospital Luedenscheid, Maerkische Kliniken, Luedenscheid, Germany

^26^Department of General Paediatrics, Medical University Graz, Austria

^27^Department of Paediatrics, Schwarzwald-Baar-Hospital, Villingen-Schwenningen, Germany

^28^Department of Paediatrics and Adolescents Medicine, University Hospital Erlangen, Germany

^29^Department of Pediatrics and Adolescent Medicine, Medical University of Salzburg, Austria

^30^Paediatric Intensive Care Unit, Medical University Graz, Austria

^31^Dr. von Hauner Children's Hospital, Ludwig-Maximilians- Universitaet, Munich, Germany

^32^Mother and Child Health Care Institute of Serbia, Belgrade, Serbia

^33^Department of Pediatric and Adolescence Surgery, Division of Pediatric Orthopedics, Medical University Graz, Austria

^34^Department of Pediatrics, Academic Teaching Hospital, Landeskrankenhaus Feldkirch, Austria

^35^University Children’s Hospital, University of Cologne, Germany

^36^Department of Pediatrics and Adolescent Medicine Wilheminenspital, Vienna, Austria

^37^Department of Pediatric Surgery, Municipal Hospital Karlsruhe, Germany

^38^Hospital of the Sisters of Mercy Ried, Department of Pediatrics and Adolescent Medicine, Ried, Austria

^39^Hospital St. Josef, Braunau, Austria

^40^Christophorus Kliniken Coesfeld Clinic for Pediatrics, Coesfeld, Germany

^41^Department of Paediatrics, University Hospital Krems, Karl Landsteiner University of Health Sciences, Krems, Austria

^42^Children‘s Hospital, Affiliate of Vilnius University Hospital Santariskiu Klinikos, Lithuania

Funding:

- Department for Science and Research of the Styrian federal government (Austria), GZ: Abt.08 -16.K-8/2012-20
- ESPID grant 2011 for "Endowed professorship for paediatric infectious diseases paying particular attention to meningococcal disease at the Department of General Paediatrics of the Medical University of Graz".
